# The Added Benefits of Performing Liver Tumor Ablation in the Angiography Suite: A Pictorial Essay of Combining C-Arm CT Guidance with Hepatic Arteriography for Liver Tumor Ablation

**DOI:** 10.3390/cancers17142330

**Published:** 2025-07-14

**Authors:** Niek Wijnen, Khalil Ramdhani, Rutger C. G. Bruijnen, Hugo W. A. M. de Jong, Pierleone Lucatelli, Maarten L. J. Smits

**Affiliations:** 1 Department of Radiology and Nuclear Medicine, University Medical Center Utrecht, 3584 CX Utrecht, The Netherlands; 2 Interventional Radiology Unit, Department of Diagnostic Medicine and Radiology, UOC Radiology, Sapienza University of Rome, 00185 Rome, Italy

**Keywords:** C-arm CT, CT hepatic arteriography (CTHA), embolization, liver tumor ablation, thermal ablation

## Abstract

The HepACAGA (Hepatic Arteriography and C-arm CT-Guided Ablation) technique, which integrates C-arm CT guidance with transcatheter C-arm CT hepatic arteriography (C-arm CTHA), significantly improves liver tumor ablation outcomes by enhancing tumor visualization, navigation, and the intraprocedural assessment of ablation margins. The two key advantages of using C-arm CT over conventional CT for image guidance are firstly that the entire procedure can be performed in the angiography suite, eliminating the need for patient transfer between the angiography suite (catheterization) and CT-room (ablation), and secondly, that integrated C-arm needle guidance software can greatly reduce the difficulty of needle placement. Beyond these advantages, the HepACAGA technique offers additional benefits across four domains: (1) the direct conversion of ablation to intra-arterial liver-directed therapies (e.g., radioembolization or chemoembolization) upon the intraprocedural detection of disease progression; (2) the direct combination of ablation with intra-arterial treatments or portal vein embolization in one session; (3) the enhanced ablation effect through heat sink effect reduction with adjunct bland embolization or balloon occlusion; and (4) the immediate hemorrhage control through direct embolization. This pictorial essay demonstrates the advantages of combining C-arm CT guidance with real-time C-arm CTHA in the percutaneous thermal ablation of liver tumors, with clinical cases illustrating each of the aforementioned four key domains.

## Simple Summary

Image-guided thermal ablation has become a cornerstone of liver cancer treatment and continues to evolve rapidly. The HepACAGA (Hepatic Arteriography and C-Arm CT-Guided Ablation) technique integrates C-arm CT navigation for precise needle placement with intra-arterial contrast administration during C-arm CT to enhance the visualization of the tumor and ablation zone. The purpose of this pictorial essay was to illustrate, through representative clinical cases, the additional advantages of performing liver tumor ablation in the angiography suite: (1) the ability to easily switch to alternative endovascular treatments (e.g., radioembolization or chemoembolization) when necessary; (2) the option to combine ablation with other endovascular therapies in a single session; (3) improved ablation efficacy by mitigating the heat sink effect through selective intra-arterial vaso-occlusive techniques, such as bland embolization with polyvinyl alcohol particles or balloon occlusion; and (4) the immediate control of post-ablation hemorrhage.

## 1. Introduction

Image-guided percutaneous thermal ablation for liver tumors is a rapidly evolving field that has become an integral part of oncological care [1,2]. Selective intra-arterial contrast injection combined with CT imaging, known as transcatheter CT hepatic arteriography (CTHA), has emerged as a promising approach for liver tumor ablation and has significantly improved ablation outcomes [3,4,5]. This method involves the catheterization of the hepatic artery to enable small-volume intra-arterial contrast injection (10–40 mL). Compared to CT-guided ablation with intravenous contrast, CTHA offers superior differentiation between the tumor and surrounding parenchyma, enhances ablation zone visualization, and requires significantly less contrast agent [6]. However, a notable drawback of traditional CTHA is the necessity for patient transfer between the angiography suite (where catheterization is performed) and the CT room (where the ablation is conducted) in centers without a hybrid angiography–CT setup. This transfer increases the risk of catheter dislodgment and contamination [7]. Also, logistical challenges arise, as both rooms and dedicated technicians need to be booked.

The HepACAGA technique (Hepatic Arteriography and C-arm CT-guided Ablation) integrates C-arm CT guidance with C-arm CT hepatic arteriography (C-arm CTHA) [7,8,9]. Utilizing C-arm CT for ablation guidance allows the entire procedure to be performed in the angiography suite, obviating the need for patient transfer and addressing the logistical challenges associated with CTHA. Additionally, C-arm systems are in many cases equipped with integrated navigation software (e.g., XperGuide by Philips or syngo Needle Guidance by Siemens), which allows for precise needle placement, even out of the axial plane. For example, subdiaphragmatic lesions in the liver dome can be approached using caudo-cranial angulation, avoiding the need to traverse the lungs.

The objective of this pictorial essay was to explore the additional benefits of performing liver tumor ablation in the angiography suite using a combination of C-arm CT guidance with real-time C-arm CT hepatic arteriography (i.e., the HepACAGA technique). To this end, we delineate four key domains of added value, each illustrated through representative clinical cases.

## 2. Technical Aspects

This pictorial essay presents six clinical cases: five were performed at the University Medical Center (UMC) Utrecht, and one case (thermal segmentectomy) was conducted at Sapienza University of Rome. All procedures were performed entirely within the angiography suite, utilizing C-arm guidance and the placement of an intra-arterial catheter for C-arm CTHA.

### 2.1. HepACAGA Procedure

**Figure 1:**
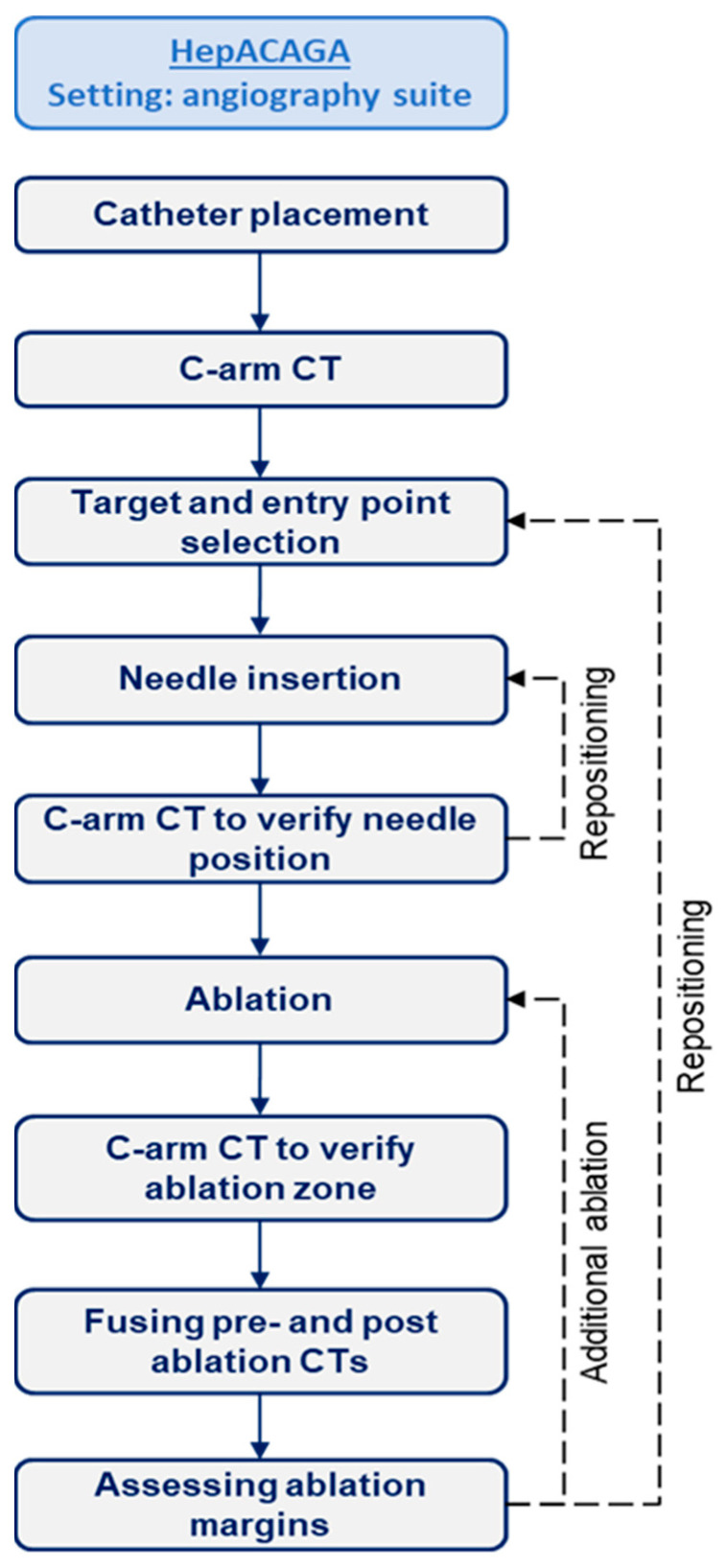
A flowchart of procedural steps involved in the HepACAGA technique.

The UMC Utrecht cases followed the HepACAGA protocol as previously described [7]. A flowchart outlining the procedural steps is provided in Figure 1. In short, all procedures were performed under general anesthesia. A catheter was introduced into the hepatic artery via femoral or radial access to facilitate the intra-arterial pump injection of small amounts of contrast agent (Visipaque 320 mg/mL, 2:1 dilution with NaCl, flow rate 0.5–2.0 mL/s, total volume 10–40 mL) for C-arm CTHA. Apnea was induced during all C-arm CTHA acquisitions to mimic the position of the liver across scans.

First, the target lesion was identified on C-arm CTHA, and a needle trajectory was planned using C-arm integrated navigation software (XperGuide, Philips, Best, The Netherlands). For hypovascular tumors lacking arterial enhancement, an additional delayed-phase C-arm CT (30–40 s delay) was acquired to improve lesion visibility. The antenna was then percutaneously advanced under real-time fluoroscopic guidance, with apnea induced to replicate liver positioning from the planning C-arm CTHA. Ablation was initiated after the confirmation of correct antenna placement on C-arm CTHA. All ablations were performed with microwave ablation (MWA) and conducted with the Emprint MWA system (Emprint^®^ HP, Medtronic, Dublin, Ireland).

Directly post ablation, another C-arm CTHA was acquired and automatically fused with a pre-ablation C-arm CTHA using the XperGuide software (https://www.usa.philips.com/healthcare/product/HCOPT06/xperguide-live-3d-needle-guidance [accessed on 2 June 2025]) to assess whether the ablation zone adequately encompassed the tumor with sufficient ablation margins. Additional ablation was performed if the margins were deemed inadequate. Finally, tract ablation was performed during antenna withdrawal to minimize the risk of bleeding or tumor seeding.

### 2.2. Thermal Segmentectomy

The thermal segmentectomy case from the Sapienza University of Rome was performed as previously detailed [10]. In summary, under local anesthesia, a catheter was placed in the hepatic artery to enable C-arm CTHA (Visipaque 350 mg/mL, 3:7 dilution with NaCl, flow rate 3.0–5.0 mL/s). Digital subtraction angiography (DSA) was performed to identify the tumor-feeding arteries and determine the optimal position for the balloon microcatheter (Occlusafe, Terumo Europe NV, Leuven, Belgium) to occlude arterial blood flow to the lesion. Selective catheterization was achieved by positioning the balloon microcatheter in the vessel proximal to all tumor feeders, with invasive arterial pressure measured at the tip of the microcatheter.

For all C-arm CTHA acquisitions, the patient was instructed to hold their breath to minimize motion artifacts. The optimal needle trajectory was planned using integrated C-arm navigation software (syngo Needle Guidance, Siemens, Forchheim, Germany, https://www.siemens-healthineers.com/en-us/angio/options-and-upgrades/clinical-software-applications/syngo-iguide [accessed on 2 June 2025]), and the microwave antenna (Amica, HS, Rome, Italy) was placed percutaneously under local anesthesia. Once correct needle placement was confirmed, the balloon was inflated to occlude arterial blood flow, and arterial pressure was measured again at the tip. Upon the detection of a drop in arterial stump pressure, balloon-occluded MWA (b-MWA) was initiated. Track ablation was performed during antenna withdrawal to reduce the risk of bleeding or tumor seeding.

With the balloon still inflated, balloon-occluded transarterial chemoembolization (b-TACE) was subsequently performed using 100 μm drug-eluting microspheres (LifePearl™, Terumo Europe NV, Leuven, Belgium) loaded with 50 mg doxorubicin.

## 3. Key Domains of Additional Benefits of HepACAGA

**Figure 2:**
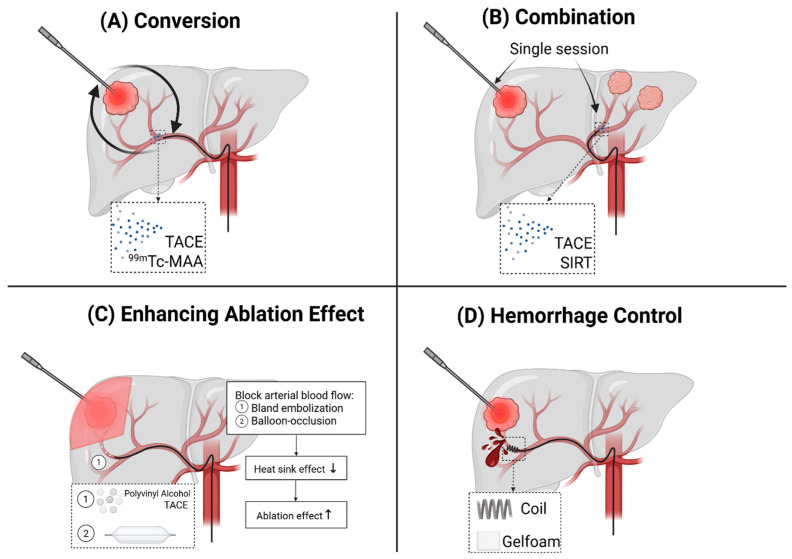
Schematic overview of the four domains representing the additional benefits of the HepACAGA technique. (**A**) Easy conversion between thermal ablation and intra-arterial therapies upon intraprocedural detection of disease progression, including transarterial chemoembolization (TACE) or administration of technetium-99m-labeled macroaggregated albumin (^99m^Tc-MAA) as part of the work-up for yttrium-90 selective internal radiation therapy (SIRT); (**B**) Combined treatment involving thermal ablation and additional endovascular therapies (TACE, SIRT, or portal vein embolization) in a single session; (**C**) Enhancement of ablation effect through adjunct techniques that reduce the heat sink effect, such as bland embolization with polyvinyl alcohol particles or balloon occlusion using a balloon microcatheter; and (**D**) Immediate post-ablation hemorrhage control through direct embolization with coils or gelfoam.

In addition to the aforementioned advantages of eliminating patient transfers between the angiography suite (catheterization) and the CT room (ablation), and the integrated C-arm navigation software that significantly reduces the complexity of needle placement, we identified four key domains where the integration of C-arm CTHA with C-arm CT guidance offers additional benefits: (1) the direct conversion of ablation to other intra-arterial liver-directed therapies (e.g., radioembolization or chemoembolization); (2) the direct combination of ablation with adjunct endovascular treatments in one session; (3) the enhanced ablation effect through heat sink effect reduction; and (4) immediate hemorrhage control. Figure 2 presents a schematic overview of these four domains. The following sections will describe and illustrate each domain with representative clinical cases.

### 3.1. Domain A—Conversion from Ablation to Intra-Arterial Treatment

Significant intrahepatic disease progression can occur between pre-ablation baseline imaging—used during tumor board meetings to determine the optimal treatment strategy—and intraprocedural imaging.

Consequently, at the start of the procedure, the interventional radiologist may conclude that thermal ablation with adequate margins is no longer safely possible. In such cases, other transarterial liver-directed therapies, such as selective internal radiation therapy (SIRT) or transarterial chemoembolization (TACE), may present more appropriate treatment options. For SIRT, the administration of technetium-99m-labeled macroaggregated albumin (^99m^Tc-MAA) can be performed as part of the work-up for yttrium-90 (^90^y) radioembolization. The ^99m^Tc-MAA can be prepared and delivered within 20 min.

**Figure 3:**
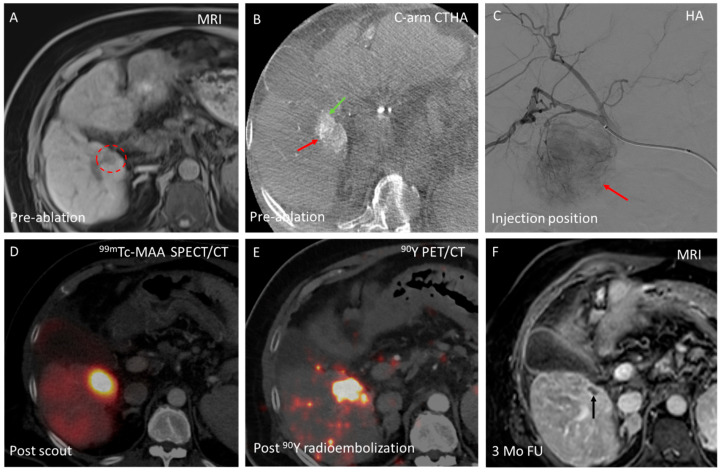
Example of conversion from an initially planned thermal ablation to a radioembolization procedure in a 55-year-old patient with a solitary HCC in segment 5. (**A**) The HCC (18 mm) (red circle) is visualized on MRI (T1 + gadolinium) 2 months before the planned ablation procedure; (**B**) On intraprocedural C-arm CT hepatic arteriography (C-arm CTHA), significant tumor growth (from 18 to 39 mm) was observed, with direct contact between the tumor (red arrow) and the gallbladder (green arrow), making ablation unfavorable. Consequently, the decision was made to switch to radioembolization and perform a ^99m^Tc-MAA scout procedure; (**C**) The tumor (red arrow) visualized on hepatic angiography (HA), with the intra-arterial catheter advanced to the right hepatic artery for injection of ^99m^Tc-MAA; (**D**) Post-scout ^99m^Tc-MAA SPECT/CT (on the same day) shows adequate tumor targeting; (**E**) Three weeks after the scout procedure, radioembolization with 570 MBq yttrium-90 (^90^Y) glass microspheres (estimated tumor absorbed dose: 500 Gy) was performed at the same injection position. ^90^Y PET/CT the following day demonstrated intense activity buildup in the target lesion, with no extrahepatic depositions; and (**F**) Contrast-enhanced MRI at 3-month follow-up showing complete response (black arrow).

With the HepACAGA technique, the entire procedure is performed in the angiography suite with an intra-arterial catheter already in position. This allows the interventional radiologist to easily switch from the initially planned thermal ablation to radio- or chemoembolization, thereby avoiding treatment delays that could lead to further disease progression. An example of a conversion of thermal ablation to SIRT is provided in Figure 3.

### 3.2. Domain B—Single-Session Combination of Ablation with Endovascular Treatment

Percutaneous thermal ablation using the HepACAGA technique enables efficient combination with other endovascular liver-directed therapies within a single session. Since the HepACAGA technique requires an intra-arterial catheter for C-arm CTHA guidance during ablation, it facilitates combination with liver-directed therapies that also rely on intra-arterial access, such as SIRT and TACE. This can be particularly beneficial for patients with multifocal liver disease, where radiation lobectomy of one liver lobe needs to be paired with ablation of lesion(s) in the contralateral lobe. Additionally, it proves valuable for patients with both lesions that are better targeted by SIRT or TACE (e.g., hypervascular lesions prone to ineffective ablation due to the heat sink effect, or lesions near critical non-target structures) and lesions that are more effectively treated with thermal ablation (such as hypovascular lesions not well targeted by SIRT or TACE).

**Figure 4:**
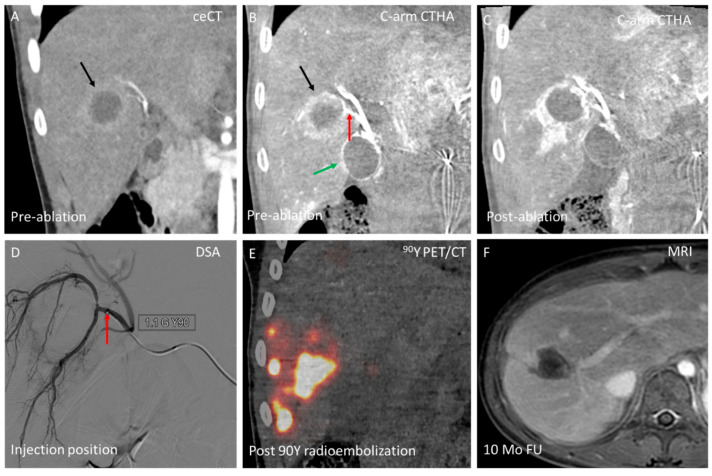
Combination of ablation and radioembolization for liver metastasis (30 mm) in segments 5/8 from a pancreatic neuroendocrine tumor (insulinoma, grade 3) in a 13-year-old patient. (**A**) Coronal view of the lesion (black arrow) on contrast-enhanced CT (ceCT) during the arterial phase, 1 month before ablation procedure; (**B**) Intraprocedural C-arm CT hepatic arteriography (C-arm CTHA) shows the lesion (black arrow) in close proximity to the gallbladder (green arrow) and with a selective tumor feeding artery (red arrow); (**C**) Post-ablation C-arm CTHA shows intentionally tight ablation zone (debulking ablation) to prevent collateral damage to gallbladder and central bile ducts; (**D**) Digital subtraction angiography (DSA) illustrates advancement of the intra-arterial catheter to the tumor feeding artery in segments 5/8 (red arrow, same as in panel B) for superselective injection of 1.1 GBq yttrium-90 glass microspheres (estimated tumor absorbed dose: 500 Gy). This was followed by a non-selective injection into the right hepatic artery to treat additional small lesions scattered throughout the right lobe; (**E**) ^90^Y PET/CT the following day demonstrated intense activity buildup in the target lesion; and (**F**) MRI (T1 + gadolinium) at 10-month FU demonstrated complete response.

In certain cases, combining SIRT or TACE with ablation for the same lesion can be advantageous. For example, when a (large) lesion is located near a critical non-target structure that cannot be separated using hydro- or pneumodissection (e.g., central bile ducts or gallbladder), debulking ablation combined with selective SIRT or TACE can effectively treat the tumor while minimizing the risk of collateral damage. An example of combined ablation and SIRT for the same lesion is shown in Figure 4.

In addition to combining the HepACAGA technique with other transarterial liver-directed therapies, it is also possible to integrate percutaneous thermal ablation with portal vein embolization (PVE) in a single session. For patients requiring liver surgery but having insufficient future liver remnant (FLR) function, increasing FLR volume before surgery is crucial. This can be achieved through PVE, in which the portal blood flow to the liver segments that will be resected is blocked by embolization, while redirecting portal flow to the FLR segments to promote hypertrophy.

**Figure 5:**
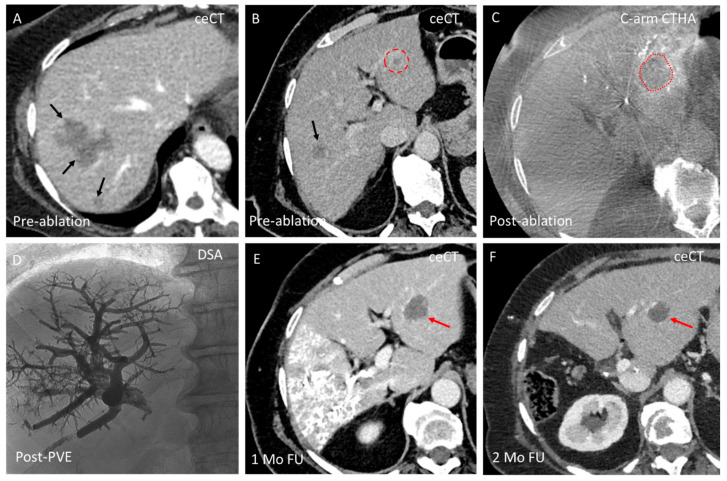
Combined thermal ablation and PVE in a 72-year-old patient with multiple colorectal liver metastases (CRLMs) in the right liver lobe and a 9 mm CRLM in segments 2/3. The patient required a two-stage liver resection: first, inducing future liver remnant (FLR) hypertrophy through PVE of the right portal vein branches, followed by right hemihepatectomy. To prevent progression of the subcentimeter lesion in the FLR, PVE was combined with ablation of this lesion. (**A**,**B**) Axial contrast-enhanced CT (ceCT) images, two months before the ablation procedure, show multiple lesions (black arrows) in the right liver lobe and a subcentimeter target lesion in segments 2/3 (red circle); (**C**) Post-ablation C-arm CT hepatic arteriography (C-arm CTHA) confirms an adequate ablation zone (outlined in red) after 6.5 min at 100 W; (**D**) Following ablation, the portal vein was percutaneously punctured under ultrasound guidance. A mixture of Glubran 2 and lipiodol (1:8) was injected, successfully occluding all portal vein branches in segments 5 to 8; (**E**) One-month follow-up (FU) ceCT confirms the desired outcome of the combined procedure: embolization of portal vein branches in segments 5–8 (visible due to the Glubran–lipiodol mixture) has led to FLR hypertrophy. Compared to the pre-ablation ceCT (panel B), the left liver lobe (FLR) has become visibly enlarged. Additionally, the ablation zone (red arrow) in segments 2/3 is visualized, with no signs of residual or recurrent tumor; and (**F**) Two-month FU ceCT shows the liver remnant, including the ablation zone (red arrow), after successful right hemihepatectomy.

Patients with one or more lesions in the FLR may require ablation or resection to ‘clean the FLR’ prior to PVE. If the ablation is performed in the angiography suite, it is possible to combine PVE and percutaneous thermal ablation in a single session. This approach is resource and time efficient, preventing delay and potential tumor progression between procedures and meaning that patients only need to be admitted to the hospital once. Figure 5 illustrates a clinical case of a combined ablation and PVE.

### 3.3. Domain C—Enhancing Ablation Effect

**Figure 6:**
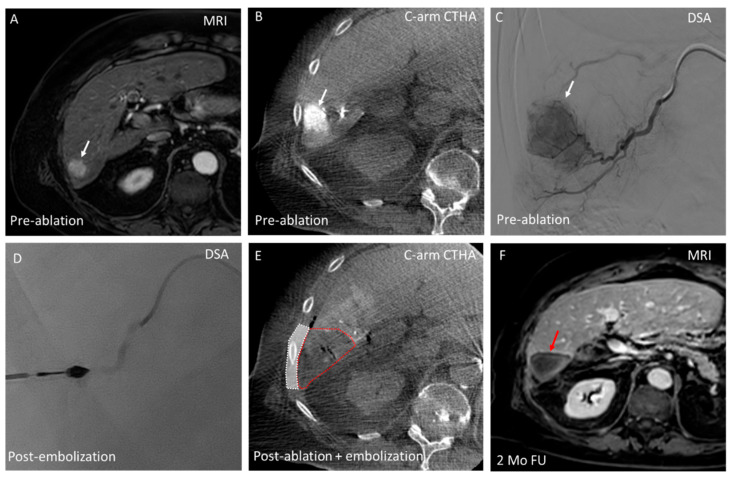
Combined bland embolization and ablation in a 55-year-old patient with a solitary HCC in liver segment 6. (**A**) The HCC (26 mm) (white arrow) is visualized on MRI (T1 + gadolinium) 2 months prior to the HepACAGA procedure; (**B**) Intraprocedural C-arm CT hepatic arteriography (C-arm CTHA) identifies the subcapsular lesion (white arrow) in close contact with the abdominal wall; (**C**) Digital subtraction angiography (DSA) demonstrates the target lesion (white arrow) and selective catheterization of the tumor-feeding artery using a 2.4 F Progreat microcatheter. PVA particles (150–250 µm) were injected to mitigate the heat sink effect before ablation; (**D**) Post-embolization DSA reveals contrast stasis in the tumor-feeding artery, confirming effective arterial occlusion prior to ablation; (**E**) Post-ablation C-arm CTHA confirms an adequate wedge-shaped ablation zone (outlined in red) after 5.5 min at 150 W. The adjacent abdominal wall (outlined in white) maintains its protective (heat sinking) arterial perfusion, preventing thermal injury; and (**F**) MRI (T1 + gadolinium) at 2-month follow-up (FU) demonstrates the wedge-shaped ablation zone (red arrow) with no evidence of local tumor recurrence.

The heat sink effect, a phenomenon in which blood flow through the vessels in proximity to the tumor dissipates the therapeutic heat generated during thermal ablation, comprises treatment efficacy and increases the risk of incomplete tumor ablation [11,12]. To mitigate the heat sink effect, selective intra-arterial vaso-occlusive techniques can be performed prior to the ablation to block the blood flow through tumor-feeding arteries. This can be performed with bland embolization, which encompasses the injection of polyvinyl alcohol (PVA) embolic particles to occlude tumor feeding arteries [13]. In practice, it is advised to first place the ablation antenna in the tumor and then inject the PVA particles selectively into the tumor feeding artery. Ablation is initiated as soon as there is stasis of arterial flow. The goal can be (1) to achieve an overall larger ablation zone; or (2) to protect nearby non-target structures. An example of protecting nearby non-target structures is shown in Figure 6. By blocking the arterial flow in the target liver segment, the ablation zone extended to a larger, wedge-shaped, confined zone, whilst relatively sparing the abutting abdominal wall that retains its protective (heat sinking) arterial flow.

**Figure 7:**
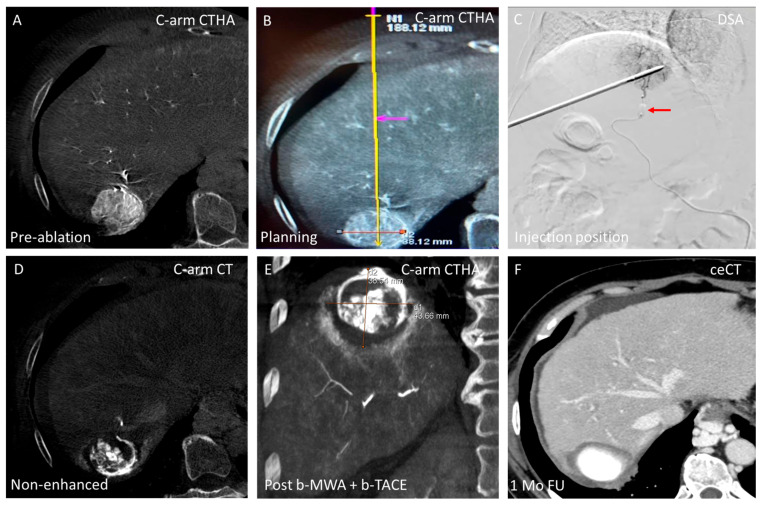
Balloon-occluded MWA followed by balloon-occluded TACE—thermal segmentectomy. (**A**) Arterial C-arm CT showing a subdiaphragmatic hepatocellular carcinoma (38 mm); (**B**) Needle trajectory planning using integrated C-arm navigation software (syngo Needle Guidance, Siemens); (**C**) Digital subtraction angiography (DSA) showing the microwave antenna positioned within the tumor and superselective microcatheter with inflated balloon (Occlusafe, Terumo) (red arrow) occluding arterial flow to the target lesion. Following an arterial stump pressure drop (measured at catheter tip), b-MWA was initiated. Subsequently, b-TACE was performed under continued balloon occlusion using 100 μm drug-eluting microspheres (LifePearl™, Terumo) loaded with 50 mg doxorubicin; (**D**) Immediately following thermal segmentectomy (b-MWA + b-TACE), a non-enhanced C-arm CT was acquired to identify microbead deposition in the target lesion through contrast entrapped within the beads; (**E**) Coronal C-arm CTHA (venous phase) confirming complete lesion coverage with adequate ablation margins; and (**F**) Contrast-enhanced CT (ceCT) at 1-month follow-up (FU) demonstrating complete response.

Another approach to enhance the ablation effect involves first blocking the arterial blood flow to the tumor with balloon occlusion using a balloon microcatheter, followed by either ablation alone (b-MWA) or ablation combined with subsequent balloon-occluded TACE (b-MWA + b-TACE)—a technique referred to as thermal segmentectomy [10]. An example of a b-MWA plus b-TACE is shown in Figure 7.

For optimal effectiveness, embolic particles or balloon occlusion should be applied immediately prior to ablation. Consequently, combining bland embolization or balloon occlusion with ablation is logistically more straightforward when the entire procedure is performed in the angiography suite with an intra-arterial catheter in place for C-arm CTHA.

### 3.4. Domain D—Hemorrhage Control

**Figure 8:**
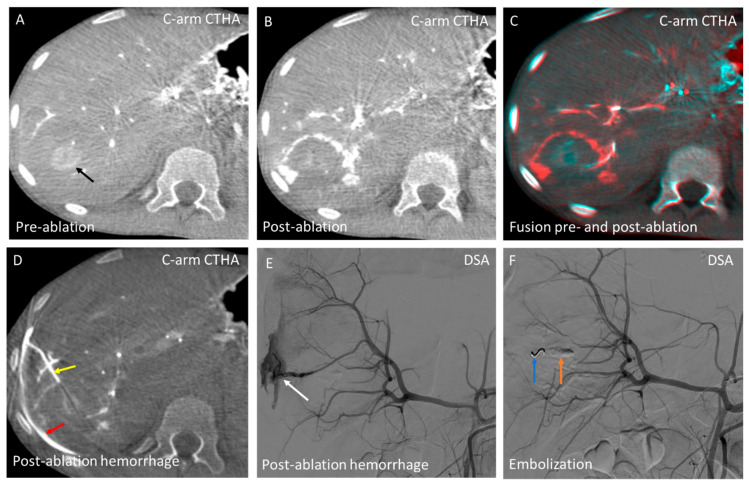
A 13-year-old patient with severe post-ablation hemorrhage requiring immediate embolization. (**A**) The target lesion (black arrow) (20 mm) is detected on intraprocedural C-arm CT hepatic arteriography (C-arm CTHA); (**B**) Post-ablation C-arm CTHA depicts the ablation zone after 3.5 min at 150 W; (**C**) Axial fusion of pre- and post-ablation C-arm CTHA shows adequate ablation margins; (**D**) Post-ablation arterial hemorrhage is observed in the ablation needle tract (yellow arrow) with subcapsular bleeding (red arrow); (**E**) Digital subtraction angiography (DSA) reveals contrast extravasation (white arrow), requiring immediate embolization; and (**F**) Bleeding was resolved within 8 min from onset with coil (blue arrow) and gelfoam embolization (orange arrow). Post procedure, the patient remained hemodynamically stable with only temporary complaints of shoulder pain.

Another distinct advantage of performing the entire procedure in an angiography suite with C-arm CT guidance and an intra-arterial catheter for hepatic arteriography is the ability to perform immediate embolization in the event of post-ablation hemorrhage. With the intra-arterial catheter already in place, embolization can be performed without delay. Figure 8 illustrates a case with severe post-ablation arterial bleeding, where direct embolization was successfully performed. A massive hemorrhage like this could have had severe consequences if a transfer from the CT room to the angiography suite had been required, highlighting the potentially life-saving benefit of this approach.

## 4. Discussion

This pictorial essay highlights the added benefits of combining C-arm CTHA with C-arm CT guidance for thermal ablation, categorizing these benefits into four key domains: (1) conversion to (2) or combination with other endovascular treatments; (3) an enhanced ablation effect through heat sink effect mitigation; and (4) direct hemorrhage control.

Conversion to other endovascular treatments can be required in the case of significant tumor growth between baseline imaging and intraprocedural imaging, occasionally precluding safe ablation with adequate margins [14]. In addition, (C-arm) CTHA provides diagnostic value by enhancing lesion detection. In a retrospective cohort study of 152 CRLM patients, CTHA identified new lesions in 10% of cases that were occult on pre-procedural imaging. Dual-phase C-arm CT further enhances lesion detection, achieving a sensitivity of 94–99% for HCC, compared to 78–85% with pre-procedural MRI or multidetector CT [15,16]. In another retrospective cohort study, 22% (54/243) of lesions identified using dual-phase C-arm CT were not visible on pre-procedural imaging [16]. Tumor growth or the detection of previously unidentified distant intrahepatic lesions may render thermal ablation ineffective or unsafe [17]. When direct conversion to alternative transarterial therapies (TACE or SIRT) is feasible, it may prevent treatment delays that could otherwise permit further tumor progression. Avoiding such delays reduces the risk of further disease advancement and increases the likelihood of successful treatment with minimally invasive locoregional therapies, potentially improving patient outcomes [18].

Not only is conversion to other endovascular treatments important, but their combination in a single session is also valuable [19,20,21]. For instance, combining ablation with TACE or SIRT for the same lesion can be particularly beneficial for large tumors. A randomized controlled trial comparing TACE alone (20 patients) to TACE combined with ablation in a single session (30 patients) for unresectable HCC demonstrated that the combined treatment was safe and superior to TACE alone for tumors >4 cm (local tumor progression [LTP] rates of 50% vs. 75% at 6-month follow-up) [19]. The HORA EST HCC trial showed that adjuvant holmium-166 SIRT following radiofrequency ablation (RFA) is safe and technically feasible in early-stage HCC patients (n = 12; tumor size 2–5 cm), with doses of up to 120 Gy well tolerated and no recurrences observed at 1-year follow-up [22]. However, SIRT was performed 1–2 months after RFA, leaving a potential window for interval tumor progression. The HepACAGA technique, by contrast, allows for the immediate combination of ablation and SIRT within a single session. Furthermore, studies have shown that PVE—intended to induce contralateral FLR hypertrophy—can increase the risk of tumor growth in the untreated contralateral lobe due to elevated angiogenic factors [23,24]. Therefore, in cases of bilobar disease with multifocal involvement in one lobe and focal disease in the contralateral lobe, it is beneficial to combine PVE of the multifocal area with local ablation of the focal disease area in a single session.

One of the most important factors in preventing LTP is achieving adequate tumor coverage with appropriate ablation margins [25]. When a tumor is located in close proximity to a large vessel, the heat sink effect can reduce the minimal ablation margin to <5 mm, increasing the risk of LTP by 3.6-fold compared to margins ≥5 mm [26]. To mitigate the heat sink effect, we perform all HepACAGA procedures using MWA, as it is less susceptible to this phenomenon compared to RFA [27]. Nevertheless, in cases where a (large) tumor has a large tumor-feeding artery, the addition of arterial occlusion—using bland embolization or balloon occlusion—can be beneficial to increase the ablation zone [10,28]. A recent study comparing MWA alone (63 patients) with MWA combined with bland embolization (49 patients) for HCC demonstrated that the combined approach improved LTP rates (7.5% vs. 5.5%, respectively) and significantly enhanced overall survival (2-year OS: 85% vs. 71%) [13]. Performing adjunct arterial occlusion to thermal ablation is more feasible when conducted in the angiography suite, with an intra-arterial catheter already in place for C-arm CTHA (i.e., HepACAGA).

Overall, the risk of significant post-ablation hemorrhage is low (<2%); however, it remains one of the most common major complications associated with liver tumor ablation [29,30]. Venous bleeding is often self-limiting, whereas arterial bleeding may necessitate transarterial embolization [31]. The prompt embolization of arterial bleeding may prevent the need for blood transfusion and its associated risks. Although rare, mortality due to severe post-ablation hemorrhage has been documented in the literature [32,33,34]. In these studies, however, no intra-arterial catheter was in place, preventing immediate embolization. The HepACAGA technique, which enables direct hemorrhage control through embolization, has the potential to be life-saving in such cases.

Compared to conventional US-/CT-guided ablation for liver tumors, the HepACAGA technique offers several substantial benefits, including improved tumor visualization, needle navigation, and ablation margin assessment [7,8,9]. A recent comparative study demonstrated that HepACAGA significantly reduced the LTP rates (5% vs. 26%), improved LTP-free survival (1-year LTPFS: 97% vs. 80%), and lowered the overall complication rate (4% vs. 15%) compared to conventional US-/CT-guided ablation [8]. Notably, pneumothorax, the most common complication overall, was observed in 8% of conventional ablation cases, but did not occur in any HepACAGA procedures. This difference is attributed to the enhanced flexibility in needle angulation provided by C-arm guidance, which enables steep caudo-cranial trajectories and safe access to dome lesions without traversing lung tissue. While the HepACAGA procedure increased procedure time (+14 min per treated lesion), it required less contrast agent (−40 mL per treated lesion). This is considered a minor trade-off given the significant improvements in clinical outcomes.

The HepACAGA technique also enables the effective ablation of small-sized lesions (≤1 cm) that are often undetectable with US or conventional CT. In a recent study investigating subcentimeter lesions presenting as foci of diffusion restriction on MRI, all 26 lesions were successfully detected and ablated using C-arm CTHA guidance, resulting in a low LTP rate (4%) and no procedure-related complications [9]. Prior to the introduction of the HepACAGA technique, our center’s approach was often to wait and scan until these small lesions became large enough to be visible on US or CT, increasing the risk of disease progression. HepACAGA now enables timely and effective ablation for these lesions.

Beyond its clinical benefits, the HepACAGA technique also offers practical and health-economic advantages. The technique has a favorable learning curve for interventional radiologists experienced in image-guided procedures. From a cost-effectiveness perspective, HepACAGA streamlines the workflow by enabling both catheterization and ablation to be performed within the angiography suite. This eliminates the need to reserve additional resources such as a CT room, extra personnel, and associated equipment. Moreover, the significantly lower tumor recurrence rates result in fewer retreatments, which may further reduce the overall healthcare costs.

Looking ahead, several technological refinements could further enhance the HepACAGA technique. Currently, general anesthesia is required to induce apnea for mimicking liver positioning across C-arm CT acquisitions. The development of reliable motion correction algorithms could eliminate this need. Additionally, automated 3D segmentation for tumors and ablation zones could improve the intraprocedural assessment of ablation margins. Also, considerable effort is being directed toward improving the image quality of C-arm CT through advances in reconstruction algorithms and artifact reduction techniques. These improvements aim to bring C-arm CT image quality closer to that of conventional CT. This can also improve C-arm navigation, for example, by enabling better differentiation of critical structures such as bile ducts and venous vessels.

## 5. Conclusions

In conclusion, in addition to the key advantages of the HepACAGA technique for liver tumor ablation—specifically, the elimination of patient transfer and the use of C-arm integrated navigation software that significantly simplifies needle placement—this pictorial essay illustrates the additional benefits of combining C-arm CTHA with C-arm CT guidance. It allows for more effective treatment through direct conversion to other transarterial therapies in cases of significant disease progression, combination with other endovascular treatments in a single session, and the mitigation of the heat sink effect with selective intra-arterial vaso-occlusive techniques targeting arteries in proximity to the tumor. It can also enhance safety in the event of post-ablation hemorrhage by allowing immediate embolization without delay.

## Abbreviations

b-MWA, balloon-occluded microwave ablation; b-TACE, balloon-occluded transarterial chemoembolization; C-arm CT, C-arm computed tomography; C-arm CTHA, C-arm CT hepatic arteriography; ceCT, contrast-enhanced CT; CRLM, colorectal liver metastases; CT, computed tomography; CTHA, CT hepatic arteriography; DSA, digital subtraction angiography; FLR, future liver remnant; HA, hepatic arteriography; HepACAGA, hepatic arteriography and C-arm CT-guided ablation; HCC, hepatocellular carcinoma; MWA, microwave ablation; PVA, polyvinyl alcohol; PVE, portal vein embolization; RFA, radiofrequency ablation; SIRT, selective internal radiation therapy; and TACE, transarterial chemoembolization.
